# Professional socialization: an analytical definition

**DOI:** 10.18502/jmehm.v12i17.2016

**Published:** 2019-12-07

**Authors:** Homa Sadeghi Avval Shahr, Shahram Yazdani, Leila Afshar

**Affiliations:** 1 *PhD Candidate in Medical Education, School of Management and Medical Education, Shahid Beheshti University of Medical Sciences, Tehran, Iran.*; 2 *Professor, School of Management and Medical Education, Shahid Beheshti University of Medical Sciences, Tehran, Iran.*; 3 *Associate Professor, Department of Medical Ethics, School of Traditional Medicine, Shahid Beheshti University of Medical Sciences, Tehran, Iran.*

**Keywords:** Professional socialization, Concept analysis, Education, Medical students, Professionalism

## Abstract

Professional socialization is defined as a process through which a person becomes a legitimate member of a professional society. This will have a great impact on an individual’s professional conduct and morality. The aim of this study was to clarify this concept and reduce the ambiguities around it.

This was a qualitative research through which the concept of professional socialization was analyzed using Walker and Avant’s eight-step approach. The review of literature for this concept was done using electronic database without any time limitation. The overall search produced about 780 articles, and after reviewing these articles, 21 were selected purposefully.

Based on concept analysis, we propose the following analytical definition: Professional socialization is a nonlinear, continuous, interactive, transformative, personal, psychosocial and self-reinforcing process that is formed through internalization of the specific culture of a professional community, and can be affected by individual, organizational and interactional factors. This definition is in accordance with the interactionism perspective. Existence of a particular profession and getting involved in a community of practice are the antecedents of this process, and formation of professional identity and professional development are its consequences. A case model, as well as borderline and related cases, has been introduced for this concept. The results of this study can be used to design useful educational interventions to conduct and facilitate the process.

## Introduction

Socialization is the process through which individuals acquire the knowledge, skills, attitudes, values, norms and appropriate actions of their community (1). Socialization begins by learning the norms and roles of the family, subcultures and self-concept, and continues throughout a person’s whole life. By growing older and joining new groups, people will take on new roles and learn new norms, and will refine their self-concept. Professional socialization is a process that people who want to enter a particular profession must go through (2). It is part of the lifelong socialization process, although in much of the existing literature, the concepts of socialization and professional socialization have been used interchangeably (3). Professional socialization is like a journey that leads to the transition from marginal to full participation in a professional society (4). It should be noted, however, that professional socialization is different from mere education. In any profession, training is the learning of knowledge and the related skills, while socialization combines this knowledge with the changed sense of oneself (5). Professional socialization is essential for a successful academic graduation experience, and its inappropriate formation may lead to dissatisfaction and dropping out of school (6). Until the 1940s, this concept was relatively uncommon, but after World War II, it attracted the interest of many researchers and scholars of various disciplines and interdisciplinary studies and entered dictionaries and scientific works such as the Talcott Parsons theory (7). Since then, this concept has repeatedly been used in scientific literature with different terminology such as acculturation (8, 9); adaptation (10 - 13); assimilation (14); social assimilation (15); organizational socialization (16, 17); and a variety of definitions. Given the diversity in defining the concept of "professional socialization" in scientific literature and the use of different terms by researchers, policymakers and educational experts in the medical community, it would be useful to clarify this concept. Therefore, providing a good analytical definition for this concept through disambiguation could provide opportunities for improving educational programs and supporting strategies in this process. The aim of this study was analyzing the concept of professional socialization and proposing an analytical definition for it.

## Methods

Talking about concepts helps researchers of a discipline to reach consensus on their own specific perceptions and avoid using them unconsciously (18). Concept Analysis is a well-known strategy for developing an analytical definition of a concept. In this process, the concept is first decomposed into its main elements and reviewed to better define and explain its attributes, and will finally be reconstructed. Walker and Avant's approach is one of the most common methods of conceptualization and development of concepts, and is a simplified form of Wilson's classical method. This logical positivistic approach aims to develop a theory through simplifying and clarifying a concept (19). In this study, the concept of professional socialization was analyzed by using this approach, which has eight basic, continuous steps (Table 1),

**Table 1:** The Walker & Avant′s model of concept analysis (19)

	Concept selection
1	Determining the purpose of the analysis
2	Identifying the uses of the concept
3	Determining the defining attributes of the concept
4	Identifying a model case
5	Identifying the borderline, related and contrary cases
6	Identifying the antecedents and consequences
7	Defining the empirical referents

With regard to the first step, it is worth noting that despite the many researches in this field, the concept is neither well-defined nor completely understood. As for the second step, the purpose of analyzing the concept of professional socialization is determining the key features in order to clarify the meaning and provide a theoretical definition that can be used for educational purposes in the medical field. As the third step of Walker and Avant's approach, in order to identify all the scientific uses of the concept and find the defining attributes, a comprehensive electronic search without any time limitation was done (through Nov. 2017). The search strategy has been shown in Figure 1. The search for literature continued until full saturation and repetition of the data. In order to enhance the credibility of the study, the opinions of two experts in the field were used for the audit.

## Results

In total, 21 published documents including 16 articles, and 5 theses containing fairly complete information about the concept of professional socialization, were selected for analysis. The rejected articles did not meet the criteria for entering the study (English language, full text access), were repetitive, or did not provide the necessary information for concept analysis because of issues related to ethics, professionalism, and educational methods and measurements. After reviewing the selected articles, the nature, attributes and other relevant features of the concept were identified, analyzed and categorized. 

***Defining Attributes of Professional Socialization Concept***

According to Walker and Avant’s method, the characteristics of a concept are the attributes used when discussing that concept, and have a key role in differentiating it from similar ones (19). At this stage, after carefully reviewing the selected literature, all the phrases involving the attributes, sub constructs, goals, or everything revealing a certain aspect of the concept, were specified in the form of direct quotes, and then the potential definitional attributes were developed through a deductive and inductive process. After that, the necessity and sufficiency tests were done. Test of necessity indicates whether the defining attributes are essential characteristics of the concept and their elimination leads to a defect or not. By using a sufficiency test, the researcher is reassured that the entire list of defining attributes has been considered (20). In this study, six conceptual areas were identified for the concept of professional socialization: identity, attributes, verbs, contents, outcomes, and affecting factors (Table 2).

**Figure:**
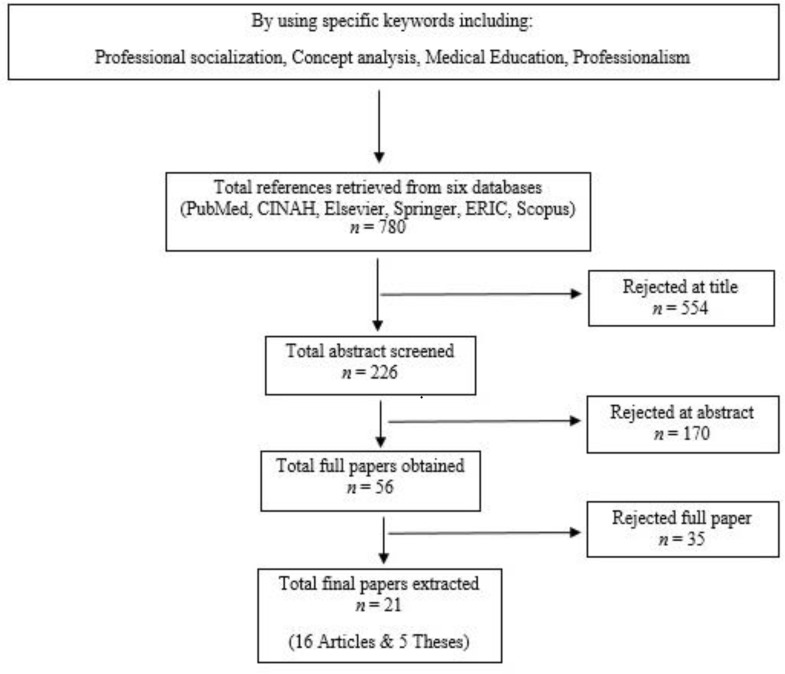


**Table 2:** Conceptual areas identified in concept analysis of professional socialization

Definition al Areas	Potential Definitional Attributes	Citation		Main PDAs
**Identity**	Process	3, 33 - 40, 43, 45, 47, 48, 50, 53, 57		Process
**Attributes**	Interactive	33, 47, 48, 50		Interactive
	Nonlinear	40, 48		Nonlinear
	Developmental	38, 40, 45, 47, 48, 53		Developmental
	Continuous	34		Continuous
	Transformative	38, 59		Transformative
	Personal	48		Personal
	Psychological	57		Psychosocial
	Social	57		
	Self-Reinforcing	30		Self-Reinforcing
**Verbs**	Internalizing	48		Internalizing
**Contents**	Culture	3, 33, 34, 38, 43, 57		Culture
**Outcomes**	Development of professional identity & Professional Development	3, 47, 48, 57		Professional Identity Development Professional Development (Later Outcomes: Job Satisfaction, Organizational Commitment, and Becoming an Effective Member of the Profession)
**Affecting ** **Factors**	Age		3, 37	Individual
	Race		33, 45	
	Gender		3, 30, 33, 37, 45	
	Family		35, 47	
	Friends		30, 43, 47	
	Experiences		3, 30, 33, 35, 36 - 38, 40, 48	
	Socioeconomic Status		36, 45, 50	
	Culture		30	
	Motivation		34, 35, 43	
	Curriculum		30, 33	Organizational
	Learning Environment		3, 48, 30, 37	
	System Structure& Climate		33, 36, 48, 30	
	Role Models		57, 36, 47, 30	
	Peers		30, 35, 40, 43, 47, 50	
	Patients		30, 36, 47, 57	
	Personnel		57	
	Mentoring		30,36, 48, 50	
	Practitioners		53	
	Others in Healthcare Roles		36, 43, 47	
	Interpersonal, Gender, Mentor & Faculty Social Interactions		3, 30, 33, 35, 37, 40, 50, 57	Interactional

***Building a Model Case***

A model case is an example that has all the defining attributes of the concept, and helps to further clarify the concept and increase its validity (20). 

Ms. “A” is a final year medical student. Ever since she was a child, she wanted to be a doctor. After admittance into a medical school, she sees herself at the beginning of a long journey, has only general and stereotypical information about the profession, and her expectations of the role are more idealistic. Early on in her studies, especially in the first and second years, she uses every moment to learn specialized terminology and content knowledge by attending the theoretical classes. She is an active and interested student, and upon entering the clinical environment, gradually becomes familiar with the power structure and the hierarchy in the profession. She tries to acquire the necessary information about the new role and adapt to it through personal observations and interactions with the professional community. Initially, through observation, participation, repetition and practice, and ultimately taking the role, she emulates what is expected of someone in the role. Over the next few years, she tries to analyze and interpret her environment to stabilize her professional goals and adjust her continuing professional growth by looking for positive feedback. She has now become a familiar face in the program and receives tacit and informal principles of the profession through communicating with faculty and peers. Now she feels more confident in the new role, and after observing and participating in some surgical processes, develops an interest in this field and decides to continue studying it. By having a positive attitude toward the role, her personality is integrated with the social structures of this role, and now she sees herself as part of the professional community.

***Alternative Cases***

According to Walker and Avant, identifying and presenting the most prominent attributes of a concept is difficult, because it may overlap with some related concepts. Examining cases that are not exactly the same as the studied concept, but are similar or contrary to it, in some ways helps the researcher to make a better understanding and assessment about attributes that have the best fit. These additional cases include: related, borderline, and contrary cases (19). In this article, however, the contrary case, which does not include any of the main attributes of the concept, and indicates what the studied concept is not (19), was not mentioned, because it does not play an important role in identifying the desired concept.

***Borderline Case***

A borderline case is an example of a concept that includes only a few, but not all, of the attributes of the concept. In this research, the concept of “organizational socialization” emerged as a borderline concept. 

Organizational socialization is a process through which a new entrant into a particular work environment acquires knowledge and skills considered by the organization and its members as essential for that particular organizational role (20).

Although there are similarities between organizational and professional socialization in certain cognitive, behavioral, ethical and emotional elements, and structural variables such as employees, the organization's power structure, role models, and important emotional experiences, these two processes are not the same. When a person chooses a profession, he/she must first enter the process of professional socialization, during which formal education is provided to individuals. Therefore, professional socialization happens prior to organizational socialization. As the next step, the person can choose an organization for employment, and that is when the process of organizational socialization begins. Entering an organization as new workforce, one tries to overcome one’s uncertainty and anxiety through seeking information and reassessment of one’s own assumptions. Professional socialization is in fact a kind of preparation that a person receives through formal education, while organizational socialization is the context in which the newcomer should start working as new workforce, and may play a more important role in shaping the performance of the newcomer compared to professional socialization (20).

An organization's field of work may often conflict with what individuals acquired through formal education, and in such cases, organizational socialization is considered more pervasive for individual development. In organizational socialization, one must be accountable to a particular organization, while professional socialization is wider and not limited to a specific organization and administration. In the latter case, the person defines oneself as a member of the profession, not a specific organization (21).

***Related Case***

A related concept is somewhat similar to the concept being studied, and therefore may be misunderstood or confused with it. Therefore, in order to prevent this, the related concept, its differences with the concept under consideration, and the degree of distinction should be determined (22).

In our study, professionalism is considered as the related concept of professional socialization. Although there are similarities between the two in terms of some attributes and definitions, they differ from each other. Professionalism is based on a social contract between the society and the members of a profession, and suggests professional behaviors that originate from professional norms. Despite the presence of similar elements such as cognitive, behavioral, emotional, moral and symbolic elements, professional socialization also has the dimension of internal adaptation, which is formed through symbolic, intellectual and psychological transformation of an individual during the process of professional socialization. In other words, the concept of professionalism is not related to independent principles such as the development of a value system and self-perception that is related to the psychological dimension of professional socialization (23).

***Antecedents and Consequences***

Antecedents are events that must exist prior to the occurrence of the concept, and identifying them can be very helpful in clarifying the areas of intervention. Consequences are the events that occur as a result of the occurrence of the concept (19).

The development of professional socialization firstly requires the existence of a particular profession as the starting point of the process. Secondly, it is necessary to get involved in a community of practice consisting of integrated educational programs, good role models, supportive educational structures, opportunities for work experience, and constructive feedback. The professional identity and professional development that are the main products of professional socialization could result in some long-term outcomes and impacts including adaptation to professional roles, job satisfaction, professional and organizational commitment, and becoming an effective member of a professional community.

***Empirical Referents***

According to Walker and Avant (19), the concepts and their attributes are abstract, and therefore cannot be good empirical indicators. Empirical referents are recognizable features of the concept that facilitate its identification and measurement, and help generate research tools. Some empirical referents for professional socialization are as follows: active participation in learning; a positive attitude to the profession (24); the students’ motivation for learning (determined by their requests for guidance and assistance in educational environments such as the skill lab); social solidarity; good rapport with colleagues and others (25); acceptable role performance (26); professional qualification approval through comprehensive qualifying exams such as 360 degree assessment and portfolio assessment; and long term outcomes including job maintenance and satisfaction.

***The Analytical Definition of Professional Socialization***

After going through the steps of concept analysis, we propose the following analytical definition of the concept of professional socialization:

Professional socialization is a nonlinear, continuous, interactive, transformative, developmental, personal, psychosocial and self-reinforcing process, which is formed in newcomers through internalizing the specific culture of a professional community, including expectations, values, beliefs, customs, traditions, and unwritten rules of the profession, as well as understanding the hierarchy and power structure, and the responsibilities. The initial and main outcomes of this process are the formation of professional identity and professional development. Various factors can affect this process, which are grouped into three categories: individual factors (gender, age, race, religion, nationality, culture, personality traits, socioeconomic status, marital status, personal experiences, and motivation); organizational factors (explicit and tacit curriculum, formal and informal learning environments, role models, and the system structure); and interpersonal relationships (interpersonal relationships with professors, peers, customers and clients, other staff, family and friends, and also receiving feedback and guided reflections).

## Discussion

***The Main Approaches to the Process of Professional Socialization***

There are two major perspectives in relation to this process in the literature: structural functionalism and symbolic interactionism (27). From the structural functionalism perspective, professional socialization is the product of newcomers acquiring the values and attitudes of a society (27). Holding this view requires adoption of a step-by-step approach to the concept, as well as acceptance of the passive and reactive behaviors of newcomers. Meanwhile, in the view known as symbolic interactionism, professional socialization is seen as a process, and interactivity is its main feature. Therefore, contrary to the previous view, here, the new entrants are actively involved in the formation of this process and play an important role in its formation and development (27).

Many studies have addressed this concept with a functionalistic approach, but a review of recently published literature in this field shows a tendency among researchers toward interactionism (27 - 31). According to the analytical definition proposed in this study, professional socialization is a highly personal process, and individual factors such as characteristics, motivations, sociocultural status, and previous experiences have powerful influences on its formation. Accordingly, the professional socialization process cannot be interpreted from the perspective of functionalism, which is described by Atkinson and cited by Ongiti (32) to consider new entrants as *tabulae rasae* (blank slates) that should be filled with essential knowledge and skills in a passive, linear, and uniform process in order to be accepted as members of a profession. On the contrary, individuals getting through this process make an active choice how to respond to the socialization process and adopt it psychologically, as viewed from the perspective of interactionism. 

***The Most Important Features of the Professional Socialization Process***

Non-linearity is an important attribute of this concept that has been mentioned in many studies (33 - 38). In these studies, features such as being cyclic (24), indirect (39), and iterative (33, 35 - 38) have also been noted, which all indicate that this process is not linear or sequential. For instance, medical students’ exposure to some events in the early stages, such as their first encounter with cadavers or death of a patient, can make major changes to this process (40). Meanwhile, there is no direct relationship between chronological time and the strength of professional socialization outcomes, and the elements of the outcomes are not all of the same weight (27).

Being cyclical means that by increasing one’s competency, one obtains more self-confidence and improves one’s capabilities through a self-reinforcing process (24). Professional socialization is a lifelong process (28, 41, 42) and does not stop when the period of formal education ends. According to the interactionism theory, the process of professional socialization can be considered as a role-taking process, which requires a continuous modification of one's role, as one enters different working environments with different facilities and challenges (43). This modification occurs to different degrees in various individuals. Therefore, the process can be seen as a highly personal experience that is dependent on the degree of one’s ability to interact with others (44), the degree of self-reflection ability (43), the composition of initial identity (45) and previous personal experiences and individual characteristics (Table2).

Most studies in this area have seen professional socialization as a developmental process (32, 34, 39, 41, 42, 46). According to Eyres, Loustau, and Ersek, as cited by Clark (43), in this process, individuals try to link the "ways of knowing" and "principles of behaving" together within a unified framework that is in accordance with the cognitive and moral developmental theories. The development of professional identity is a very important aspect of becoming a professional, and for achieving this, trainees should think, act, and feel like members of the profession (47); doing this requires individuals to negotiate with themselves, that is, to go through the stage of internal adaptation, which forms the psychological dimension of professional socialization (5). Professional socialization is a transformative process involving a symbolic, ideational and psychological transformation during which an individual’s meaning system and perspectives are transformed in order to legitimize authority to meet the public’s expectations (48). The most important attributes of this process as proposed in this study that are consistent with aforementioned literature in this areas seemed to be: transformative, developmental, continuous, and personal. 

***Professional Socialization through Internalizing Culture***

Internalizing the professional culture includes understanding and accepting the hierarchy and the power structure, responsibilities, expectations, values, beliefs, customs, traditions and unwritten rules of the profession, and occurs through formal and informal processes. The major part of formal socialization happens in basic educational programs, and the remaining part, which is often informal, occurs incidentally and subconsciously in educational and practical environments through unplanned observations and interactions with important others. The formal aspect of the process can transfer certain dimensions of professional culture, such as beliefs about the characteristics of a responsible professional who is committed to adherence to the ethical codes (49). However, transmitting the professional culture through informal situations of socialization, and the implicit effects at the organizational and structural level known as hidden curriculum, also have a significant impact on the formation of trainees' professional values and behaviors (50(. Therefore, the adoption of a new approach to this important process, through facilitating the path that leads to appropriate formation of professional identity of future professionals, can help to enhance and facilitate its formation.

***Factors Affecting Professional Socialization***

As can be seen in Table 2, several factors can influence the professional socialization process that were grouped in three categories of individual, organizational, and interactional factors. Nonetheless, all of these factors do not have the same weight in terms of impact on the process of socialization, and the roles of mentors and role models (5, 30, 39, 46, 50), previous experiences (31-34, 51), field and work experiences in the community of practice (3, 27, 31, 34, 52, 53), and reflection (5, 30, 33, 54,55, 56,57) are more prominent. Mentorship is one of the “key processes” in socializing individuals into nursing and education (58). Mentors are like the “linchpin of students’ experience in becoming socialized” who keep various elements of the practical and educational environment together, which is conducive to the students’ socialization (59).

Reflection and the students’ ability to adapt to the innate culture within the practical settings is fundamental for socialization (54). Reflection through integration of personal beliefs, attitudes and values into the professional values helps the development of professional identity (52). It seems that there is a close relationship among the three aforementioned essential elements of role modeling, personal experience and reflection. By observing role models, learners imitate and practice the role, and through guided reflection on their experience, consciously acquire effective knowledge, attitude and competence. Furthermore, students subconsciously receive the unwritten rules and culture of the profession and then internalize it consciously through self-reflection. In this manner, they ultimately will be socialized in the professional community and develop a proper professional identity.

## Conclusion

Professional socialization has been introduced in the literature using surrogate terminology such as professional preparation, professional adaptation, acculturation, assimilation and professional absorption. It is the process through which a layperson gradually becomes a professional, and adapts to a reference point for the particular values and behaviors of the role. Clarifying this concept and its elements, which was the mission of this study, can provide an important clue to future studies about the prerequisites, facilities and proper strategies for guiding students to form appropriate professional identity.

## Notes:

**Citation to this article:**

Sadeghi Avval Shahr H, Yazdani S, Afshar L. Professional socialization: an analytical definition. J Med Ethics Hist Med. 2019; 12: 17.
